# NONDERMATOPHYTIC MOULDS AS A CAUSATIVE AGENT OF ONYCHOMYCOSIS IN TEHRAN

**DOI:** 10.4103/0019-5154.62743

**Published:** 2010

**Authors:** Shahindokht Bassiri-Jahromi, Ali Asgar Khaksar

**Affiliations:** *From the Medical Mycology Department, Pasteur Institute of Iran, Iran.*

**Keywords:** *Fungal infections*, *Iran*, *mould*, *onychomycosis*

## Abstract

**Background:**

In the last few years the number of cases of nondermatophytic onychomycosis has greatly increased.

**Aim:**

To evaluate the incidence, the clinic characteristics, and predisposing factors of nondermatophytic onychomycosis.

**Materials and Methods:**

All collected specimens were analyzed by direct microscopy and culture. Microscopic examination of these specimens was carried out in potassium hydroxide solution (20%) with dimethyl sulfoxide (4%). These specimens were cultured on sabouraud's glucose agar with chloramphenicol and sabouraud's glucose agar with chloramphenicol and cycloheximide. Cultures were incubated at 25°C for up to 28 days and checked twice weekly for growth.

**Results:**

Nondermatophytic onychomycosis were 11.5% of all onychomycosis. We found that *Aspergillus spp*. were the more responsible etiologic agents of nondermatophytic onychomycosis, resulting in a total of 28 patients (59.6%). In our study other causative agents were *Acremonium spp.* (17%), *Fusarium spp*. (12.7%), *Geotrichum spp.* (4.2%), *Trichosporun spp*., (4.2%) and *Scopulariopsis spp* (2.1%). In our patients moulds onychomycosis developed mainly in toenails (74.5%).

**Conclusion:**

Knowing the exact pathogen is important and has implications in therapy and prognosis.

## Introduction

Onychomycosis refers to any fungal infections with the causative agent being dermatophytes, yeasts, and nondermatophyte moulds.[1] Onychomycosis is one of the most frequent causes of onychopathy. In Australia, UK, and USA the incidence of onychomycosis has been estimated at about 3% of the total population, increasing to 5% in older people.[2,3] Dystrophic nails harbor a large flora of saprophytes and secondarily invading fungi, yeasts, and bacteria.[4] Distorted, deformed, thickened, discolored nails with an accumulation of debris beneath them, particularly with ragged and furrowed edges, strongly suggest tinea unguium.[5] Candida onychomycosis lacks gross distortion and accumulated detritus. Leukonychia is mimicked by leukonychia of other etiologies, particularly trauma.[4]

Infections by moulds are the least frequent form and are found mainly in elderly people.[6,7] The number of patients affected by onychomycosis is continually growing and every year the number of microorganisms recognized as capable of parasitizing the nail plate directly is growing all over the world.[8] With the continued increase in the prevalence of onychomycosis, it is important to keep in mind that all isolated filamentous or pseudohyphal organisms should be evaluated as potential pathogens when diagnosing fungal infections.

The aim of this study was to determine various clinical types in our region, to isolate the causative pathogen, and to determine the etiological correlation of onychomycosis.

## Materials and Methods

From March 2005-2006 we performed nail mycology on 1268 patients with nail abnormalities. Patients were referred by dermatologists or general practitioners to our mycology service. The data collected from patients included details regarding age, sex, residence, and history of the treatment, if any. In our patients, we personally inspected the nail signs and made a presumptive clinical diagnosis before taking the samples for mycology. When the results of mycology did not confirm our presumptive diagnosis, we always reexamined the patient and took additional samples for mycology.

In patients with nail abnormalities of distal subungual onychomycosis, nail samples were obtained from the most proximal portion of the affected nail by scraping the hyperkeratotic nail bed. In proximal subungual onychomycosis we scraped the exposed nail plate with a disposable scalpel. Nail samples were microscopically studied after clearing in 20% potassium hydroxide (KOH). For cultures, nail fragments were inoculated in Sabouraud's glucose agar chloramphenicol medium, with and without cycloheximide, and incubated at 25°C for 4 weeks and checked twice weekly for any growth. Negative cultures were confirmed after 4 weeks of no growth. Twenty inoculums were performed in each plate. Slides of the culture were used for identification of the organism. Identification of fungal isolates was based on microscopic morphology. Diagnosis of nondermatophytic onychomycosis was made based on the following criteria:

1. Nail abnormalities consistent with this diagnosis;
2. Positive KOH preparation with presence of hyphae in the nail keratin;
3. Failure to isolate a dermatophyte in culture; and
4. Growth of more than five colonies of the same mould in at least two consecutive nail samplings.

The specimens that were positive by direct microscopy, but failed to grow were cultured repeatedly every time the first culture showed the growth of a mould.

If a mould was isolated at first culture, its etiologic significance for a nondermatophytic onychomycosis was confirmed after the third positive culture was examined, whereas all other cases were considered to be just contaminants.

## Results

In 410 cases (190 involving toenails and 220 involving fingernails) the etiologic agents of onychomycosis were established after repeated cultural examinations.

From 410 culture-positive specimens, 47 (11.5%) yielded nondermatophytic moulds, 166 (40.5%) dermatophytes, 197 (48%) yeasts [Table 1], and 9 (2.2%) mixed (two different fungi) growth. All patients with mould onychomycosis had subungual hyperkeratosis.

**Table 1:** Distribution of causative agents of onychomycosis

Organisms	Toenail	Fingernail	Total	Percentage
Dermatophytes	131	35	166	40.5
Yeasts	24	173	197	48
Nondermatophytes moulds	35	12	47	11.5
Total positive	190	220	410	100

The average age of our patients was 46.3 years in that 34 cases were above 40 years. The youngest patient was a 15-year-old girl and the oldest patient a 72-year-old man, the first one affected in the fingernail and the second affected in the toenails. In our study, *Aspergillus spp*. was shown to be the cause of onychomycosis in 28 (6.8%) patients. Among the moulds *Aspergillus spp*. was the most frequently responsible causative agent and represented 59.6% of total nondermatophytic onychomycosis. The other isolated s*pp.* were *Acremonium* (in 17%), *Fusarium* (in 12.7%), *Geotrichum* (in 4.2%), *Trichosporun* (in 4.2%), and *Scopulariopsis* (in 2.1%). The infections were mostly localized to the toenails.

Distal subungual onychomycosis of the biggest toenail was observed in 34 cases, bilaterally in six cases, and we also found 13 cases of infections localized in the fingernails. In our study the distal subungual onychomycosis of the biggest toenail was the most frequent (72.3%). Subungual hyperkeratosis affected 6 (12.8%) cases in more than one nail and 31 (66%) cases in the big toenails.

## Discussion

Fungi cause only 50% of nail disorders,[9] and other skin conditions can mimic cutaneous fungal infections. The incidence of nondermatophytic mould in the pathogenesis of onychomycosis is variable in epidemiologic studies. The present study reflected that 11.5% of all mycologically confirmed cases of onychomycosis were due to nondermatophytic fungi. Frequencies of mould onychomycosis in European countries like Austria,[10] Estonia,[11] Italy,[12] and Spain[13] are reported as about 5 (mean), 7, 8, and 17.2%, respectively. The prevalence in North America is 4.3% in Canada[14] and 20% in the United States,[15] whereas in South America 4.5 and 9.5% in two different centers in Colombia[16] and 1% in Argentina.[17] In Asia the frequencies are given as 12% in Singapore[18] and 22% in India.[19] A study on onychomycosis from Turkey[20] reported the prevalence of 2.1%, but the study was retrospective with no repeat cultures carried out.

Nondermatophytic moulds may be recovered as contaminates from glabrous skin, hair, and nails. Stringent criteria must be met before a nondermatophytic mould grown from a specimen is accepted as a causative agent. According to English[21] a nondermatophytic mould growth from at least 5 of 20 nail fragment inoculums, with no dermatophyte growth, is considered to be clinically significant.

Diagnosis of onychomycosis caused by moulds must be rigorous because of their frequent presence in the environment as contaminants, and is based on the criteria of English: after the first isolation, direct microscopic observation and culture must be repeated two more times.[21] Summerbell, Kane, and Krajden[22] added the presence in direct microscopy of atypical hyphae and/ or conidia to the list of criteria. In our study, the criteria were met with the presence in microscopy of atypical hyphae with repeated isolation of pure cultures of saprophytic moulds.

If a mould is demonstrated in the culture, its role can be evaluated only under the following circumstances:

1. No dermatophytic growth in the culture;
2. 5-10 of 20 nail inoculums from various samples have produced the same pathogen in sabouraud medium; and
3. The presence of the fungus in the keratin has been confirmed by both direct and, in particular, histological examinations.

The association of direct microscopy, culture, and histological examination will grant a maximum of positive results.[23] Moulds preferably invade the nails on the big toes, especially in subjects above 60 years. This can be attributed to local environmental conditions, peripheral circulatory disturbances, or specific anatomic conditions.[24]

There is an ever-growing list of moulds which have been found actively colonizing nails. It includes the comparatively common *Aspergillus spp., Acromonium spp*., and *Scopolariopsis spp.* None of these fungi are keratinolytic. Therefore, all must either live on the unkeratinized intercellular cement, or must take advantage of partial denaturing of the nail keratin by pre-existing trauma or disease.[25]

In our patients, mould onychomycosis developed mainly in toenails (74.5%). As with dermatophyte infections, mould infections are much more common in toenails than in fingernails. The infection was more prevalent in females (72.3%) than in males (27.7%). Reports show that mould onychomycosis is more frequent in toenails than in fingernails and in the adults, especially those above 50 years.[26–30]

This situation can be prone due to more traumas with age and footwear, and corresponds to very slow growth of finger and toenail plates as well to a much higher incidence of an impaired blood supply to the extremities.

The frequency according to gender is controversial.[29,31] In the present study, consistent with the data,[26–30] mould onychomycosis was more frequent in toenails than in fingernails and over age 40.

The infections by *Aspergillus spp*. represented 6.8% of all onychomycosis and 59.6% of only nondermatophytic onychomycosis are considered (of which *Aspergillus fumigatus* is 27.6% alone). *Aspergillus spp.* are a large group of common saprophytic moulds, and often isolated from soil, air, and plant materials. This group of fungi is normally considered as common contaminants in immunosuppressed patients.[32]

In all cases the distal subungual onychomycosis was localized in the toenails, especially the big toenail.

In our study the general risk factors for onychomycosis are increasing age, gender, diabetes, nail trauma, hyperhidrosis, and poor hygiene. Fungi can invade healthy nails or may invade nails previously damaged in the course of other disease (psoriasis, lichen planus, eczema) or they are altered tropically by impaired blood supply to the extremities. Damage can also be induced by hormonal disturbances (diabetes mellitus, Cushing's syndrome, hypothyroidism) or by immunosuppression (AIDS) or ongoing therapies.[33]

Moreover, the nail unit undergoes changes in old age. These changes include slower growth, abnormal maturation of corneocyte, and thickening of blood-vessel walls within the subungual area. In addition to predisposing of the nail unit is, as consequence, the efficacy of systemic antifungal treatment. Onychomycosis is mainly detected in elderly people.[34,35] Other factors which have contributed to the spreading of nondermatophytic onychomycosis are immunosuppression and the immigration phenomenon.[10,34] Recently, some authors have suggested that an untreated nondermatophytic onychomycosis could be a dangerous portal of entry for deep-seated and disseminated mycosis, that are hard to treat in immunocompromised patients.[35,36] Therefore, correct diagnosis and rapid treatment of nondermatophytic onychomycosis is indispensable.

As shown in the present study, the definite diagnosis of a mould onychomycosis is not as easy as the diagnosis of onychomycosis due to dermatophytes and yeasts. They should set up consecutive cultures from consecutive nail scrapings to reach dependable laboratory results.

Clinical and mycological criteria are important to ascertain both the diagnosis and resolution of onychomycosis.

A proper diagnosis, consisting of both clinical and mycological examinations, may aid the clinician in selecting the most appropriate therapy.

The effect of onychomycosis on patient quality of life has a significant impact.

Knowledge of epidemiological and mycological characteristics of onychomycosis has been noted by many authors as being an important tool for control of these fungal infections.
